# Analysis of risk factors associated with gas embolism and evaluation of predictors of mortality in 482 loggerhead sea turtles

**DOI:** 10.1038/s41598-021-02017-4

**Published:** 2021-11-22

**Authors:** D. Franchini, C. Valastro, S. Ciccarelli, P. Trerotoli, S. Paci, F. Caprio, P. Salvemini, A. Lucchetti, A. Di Bello

**Affiliations:** 1 Department of Veterinary Medicine, University of Bari “Aldo Moro”, SP 62 km 3, 70010 Valenzano, BA Italy; 2 Department of Biomedical Science and Human Oncology, University of Bari “Aldo Moro”, Piazza G. Cesare 11, 70124 Bari, Italy; 3 WWF Molfetta Rescue Center, via Puccini 16, 70056 Molfetta, BA Italy; 4 National Research Council IRBIM-CNR-IT, Largo Fiera della Pesca 1, 60125 Ancona, Italy

**Keywords:** Zoology, Diseases

## Abstract

Sea turtles that are entrapped in static and towed nets may develop gas embolism which can lead to severe organ injury and death. Trawling characteristics, physical and physiologic factors associated with gas-embolism and predictors of mortality were analysed from 482 bycaught loggerheads. We found 204 turtles affected by gas-embolism and significant positive correlations between the presence of gas-embolism and duration, depth, ascent rate of trawl, turtle size and temperature, and between mortality and ascent time, neurological deficits, significant acidosis and involvement of > 12 cardiovascular sites and the left atrium and sinus venosus-right atrium. About 90% turtles with GE alive upon arrival at Sea Turtle Clinic recovered from the disease without any supportive drug therapy. Results of this study may be useful in clinical evaluation, prognostication, and management for turtles affected by gas-embolism, but bycatch reduction must become a priority for major international organizations. According to the results of the present study the measures to be considered to reduce the catches or mortality of sea turtles for trawling are to be found in the modification of fishing nets or fishing operations and in greater awareness and education of fishermen.

## Introduction

Gas-embolism (GE) is the presence of gas within the cardiovascular system, which can result in a variety of systemic effects depending on the amount of gas and its anatomic distribution. In humans, GE can occur as a result of trauma, underwater diving, or as a complication of surgical and diagnostic procedures. Gas enters the circulation when a communication between a gas source and the vascular system exists together with a pressure gradient that favours gas translocation^1^. Breath-holding diving vertebrates have been considered to be protected against GE through anatomical, physiological, and behavioural adaptations^2–8^. In 2014, a study of bycaught loggerhead sea turtles (*Caretta caretta*) entrapped at depth in trawls and gillnets demonstrated that sea turtles developed gas embolism (GE)^9^. Since that time, GE has been documented in four additional sea turtle species^10,11^.

Sea turtle populations have declined dramatically in recent decades and all sea turtle species whose conservation status has been assessed, are considered to be threatened or endangered. The expansion of fishing activities in coastal areas has contributed to the decline of several sea turtle populations and currently fisheries interactions are the most serious conservation risk for threatened sea turtle populations^12^. Sea turtle interactions are known to be problematic in longline, gillnet, trawl fisheries that operate in the range of sea turtles. Bycatch is a well-documented, worldwide problem resulting in considerable mortality of non-targeted species^12–14^. A minimum of 132,000 sea turtle captures are estimated to occur annually in the Mediterranean by bottom trawlers, longlines and set nets, resulting in a minimum of 44,000 deaths, the majority owing to small-scale fisheries^15^. In particular, more than 52,000 turtles are caught yearly along the Italian coasts and about 10,000 of these die. Trawl nets are the fishing gear involving the highest bycatch probability, and this is a special concern in the Adriatic Sea, whose shallow waters are favourable for trawling and are also foraging areas rich in benthic communities for sea turtles^16^. According to several studies^17,18^, direct mortality due to trawling is correlated with trawl duration, presumably due to prolonged apnoea. Until recently, death has been attributed to the effects of hypoxia and water aspiration associated with forced submergence or to sequelae of traumatic injuries^19^. Delayed post-release mortality has been suspected to be high^16^. However, the evidence that sea turtles can also suffer from GE must be assessed as a major factor in trawling related mortality. It has been hypothesized that entrapped, submerged turtles develop DCS due to exertional activity (i.e., attempting to escape from the net), and associated catecholamine-induced sympathetic induction and parasympathetic inhibition. These effects are believed to disrupt the normal, protective vagal diving reflex that minimizes blood flow through air-filled pressurized lungs during diving^9^. Lactic acidosis and evidence of decreased renal function and cellular injury have been reported in GE-affected turtles^20^. To date, reported mortality rates due to GE in bycaught sea turtles range from 30 to 53%^9,20,21^.

The objectives of the present prospective study are: (1) to examine risk factors associated with GE in a large cohort of accidentally caught loggerhead turtles, (2) to describe the physical and physiological status in turtles with GE, (3) analysing various parameters to identify predictors of mortality, (4) to suggest a possible management of these animals once they have been caught accidentally. To define the correlations between trawling and GE we evaluated the duration and depth of trawling, the ascent rate of the fishing gear, the weight of the turtle, the length, size and body temperature of 482 sea turtles. To determine the physiological status of GE-affected turtles, and to investigate prognostic variables predictive of mortality, we also analysed respiratory rate, heart rate, presence of peripheral oedema, neurological and sensory deficits, radiographic findings, and packed cell volume (PCV) for loggerhead turtles affected by various degrees of GE. In addition, we evaluated leucocyte count (WBC), blood gas and biochemical analytes of major clinical relevance in a part of GE-affected turtles.

## Results

### Clinical and radiographic findings and outcome

Four hundred eighty-two turtles were admitted to the hospital after accidental trawl capture and were evaluated even if the animal did not show clinical signs of disease or trauma. The duration from surfacing (time of capture on fishing boat) until the animal arrived at the STC (Sea Turtle Clinic) ranged from 5 to 7 h.

**Figure 1:**
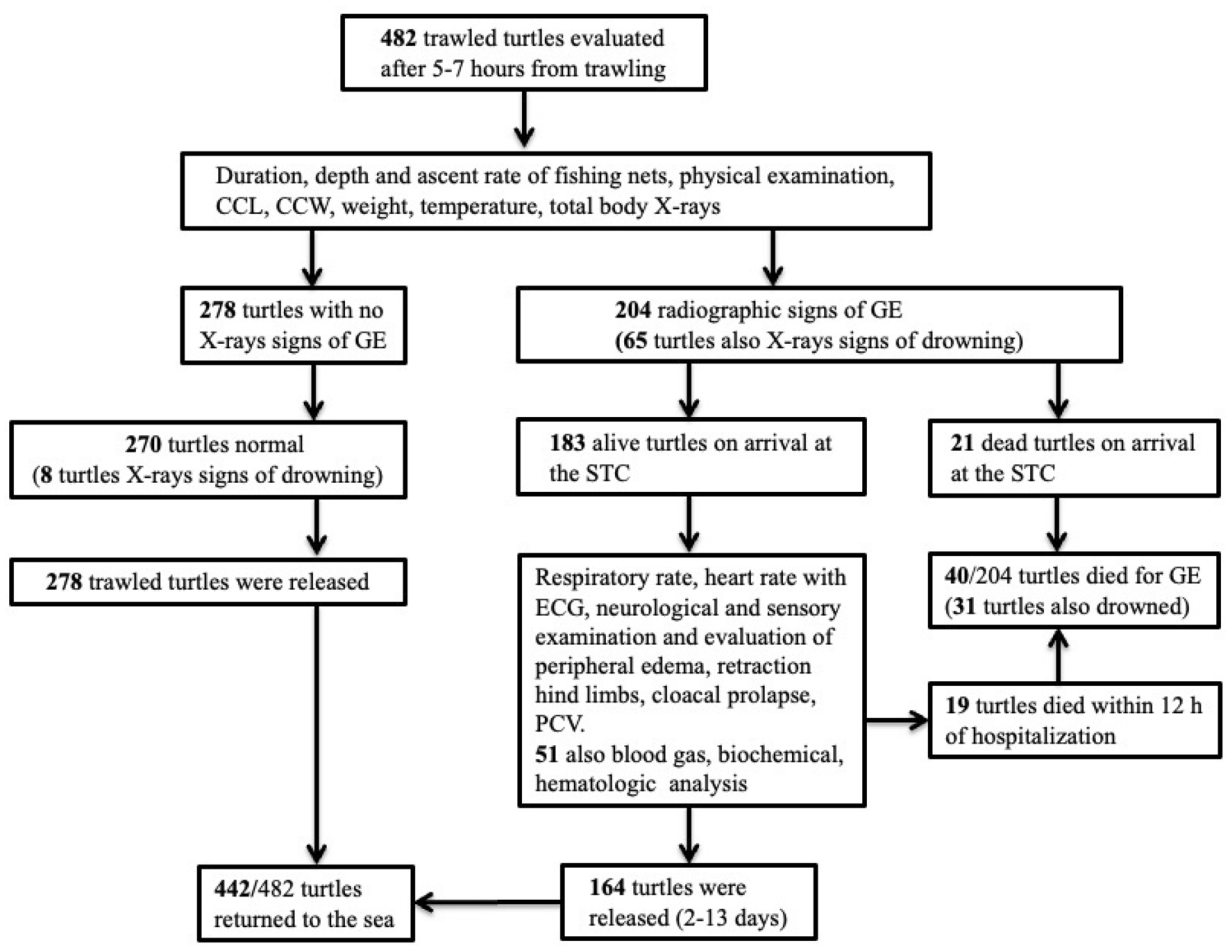
Flowchart of the outcome and clinical findings for 482 loggerhead turtles after being accidentally captured by trawling.

Turtles were mainly incidentally captured (bycaught) in trawls in the winter months, with the highest incidence in December and January. Out of 482 turtles, 308 (64%) were juveniles. Of 482 trawled turtles, 204 (42.4%) showed radiographic signs of systemic GE of variable severity (Fig. 1).

Trawl depth ranged from 11 to 144 m (median of depth 46.8 m) with a duration of fishing net submergence (soak time) ranging from 2 to 5 h (3.5 h median). The ascent rate of fishing gear was from 11 min (for a depth of 20 m) up to a maximum of 25 min (for a depth of 140 m).

Table 1 summarises the comparison of fishing and clinical data between loggerheads with and without GE.

**Table 1:** Comparison of fishing and clinical data between loggerhead turtles that were accidentally captured during trawling that developed GE (n = 204) and those that did not develop GE (n = 278).

	Turtles with GE (n = 204)		Turtles without GE (n = 278)		p-value
	Median	Interquartile range	Median	Interquartile range	
Trawl duration (h)	4	3 to 4	3	3 to 4	0.001
Trawl depth (m)	49.5	36 to 63.9	37	32 to 54	< 0.0001
Curved carapace length CCL (cm)	64	60 to 69	62	56.5 to 68	0.0096
Curved carapace width CCW (cm)	58.25	54.75 to 63	57	51.5 to 62.5	0.024
Weight (kg)	31	24.8 to 38.8	28.25	21 to 36.8	0.0278
Turtle temperature (°C)	12.5	10.9 to 14.2	13.3	11.6 to 16.6	< 0.0001

	n	%	n	%	
Male	10	4.9	16	5.8	0.2506
Female	55	27.0	93	33.5	
Immature	139	68.1	169	60.8	

Parameters with p < 0.05 were significantly different between the two groups.

The risk of GE is significantly associated with the ascent rate, in particular the ascent rate between 2.5 and 3.5 m/min (b = 0.4, p = 0.0071) and ascent rate greater than 3.5 m/min (b = 0.25, p = 0.0478) were statistically significant. Compared to turtles with an ascent rate < 2.5 m/min, the risk of GE in those with an ascent rate faster than 3.5 m/min is 2.89 (95% CI 1.65–5.03) times greater and in those with an ascent rate between 2.5 and 3.5 m/min is 2.47 (95% OR CI 1.52–4.02) times greater. Therefore, it is possible to state from the present data that the ascent rate of turtles faster than 2.5 m/min leads to an increased risk of GE.

**Figure 2:**
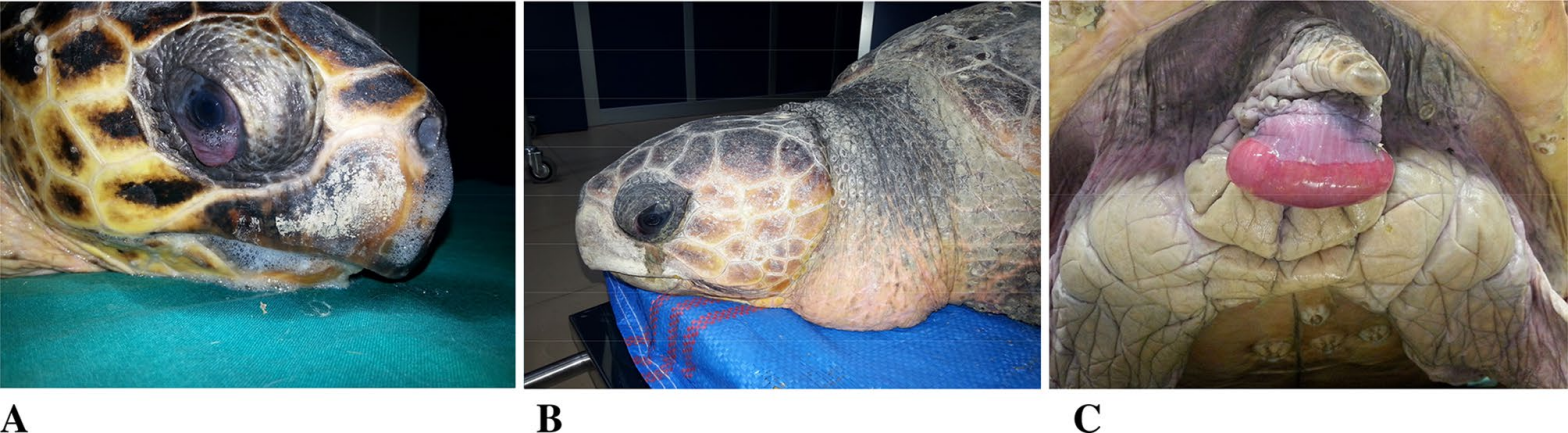
Clinical findings in turtles with GE. (**A**) Eyelid and conjunctival oedema and external foam coming from ramphotheca and nostrils in a Loggerhead turtle (*Caretta caretta*) with GE and drowning; (**B**) prominent oedema of the eyelid and neck; (**C**) prolapse of the cloaca in *Caretta caretta.*

**Figure 3:**
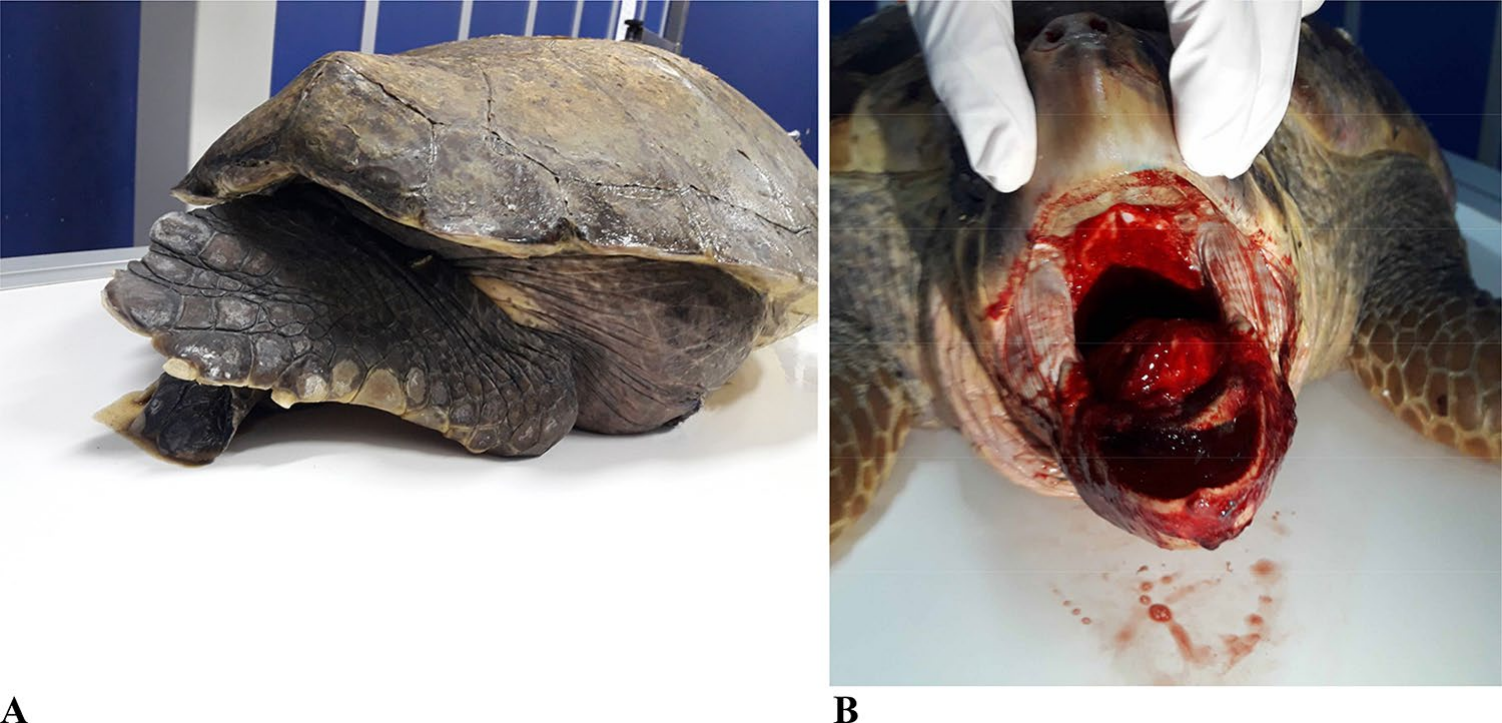
(**A**) Hind limbs flexed under the carapace in a turtle with GE; (**B**) turtle with blood coming from the oral cavity arising from the larynx that died 6 h after arriving at the STC (11 h after trawling).

On arrival, 165 (81%) of the turtles with GE presented with good body condition and normal fat stores, 19/204 (9%) were slightly thinner while 20/204 (10%) were very thin. Of the 204 turtles, 195 (95%) showed peripheral oedema and 31/204 (15%) prolapse of the cloaca (Fig. 2). These anomalies took 3–7 days to resolve. The mean respiratory and heart rates of turtles with GE were respectively 0.4 breaths/min (mean 6.4 ± 3.51 in 15 min) and 14.62 bpm (mean 14 ± 4.29) and heart rate < 12 was a predictor of mortality. At physical examination 143/183 (77%) of these turtles were alert, active, and exhibited normal behaviour, 29/183 (16%) were slightly depressed (moderate response to manipulations) and 12/183 (7%) were lethargic/comatose (no response to manipulations) or developed progressive neurological symptoms, including limb paresis or loss of nociception. 10/12 turtles of those that were lethargic/comatose died within 24 h of arrival. Seven of 184 (4%) turtles showed retraction of the hind limbs under the carapace (Fig. 3A). Three turtles had blood coming from the oral cavity arising from the larynx and died within 8 h of arrival (Fig. 3B).

Among turtles diagnosed with GE, there was no significant correlations between mortality and trawl duration, depth, animal size, sex, weight, body temperature, respiratory rate, presence of peripheral oedema and PCV while there was a significant correlation between mortality and neurological deficit, comatose state, hind limb retraction under the carapace and heart rate (Table S1). Turtles that had an ascent rate ≥ 3.5 m/min have 3.22 times greater risk of death than those with a speed < 2.5 m/min, while compared to turtles with an ascend rate between 2.6 and 3.4 m/min the risk is 2.6 times greater. The risk of death increases when the speed of 3.5 m/min is exceeded.

**Figure 4:**
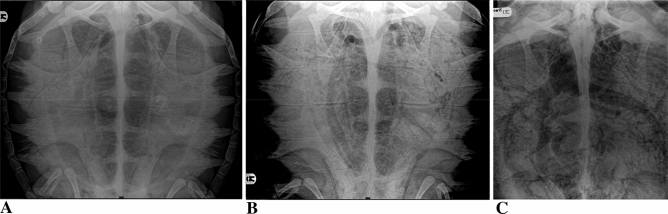
Dorsoventral radiograph of 3 Loggerhead turtles (*Caretta caretta*) with GE of mild (**A**), medium (**B**) and severe (**C**) radiographic degree.

**Figure 5:**
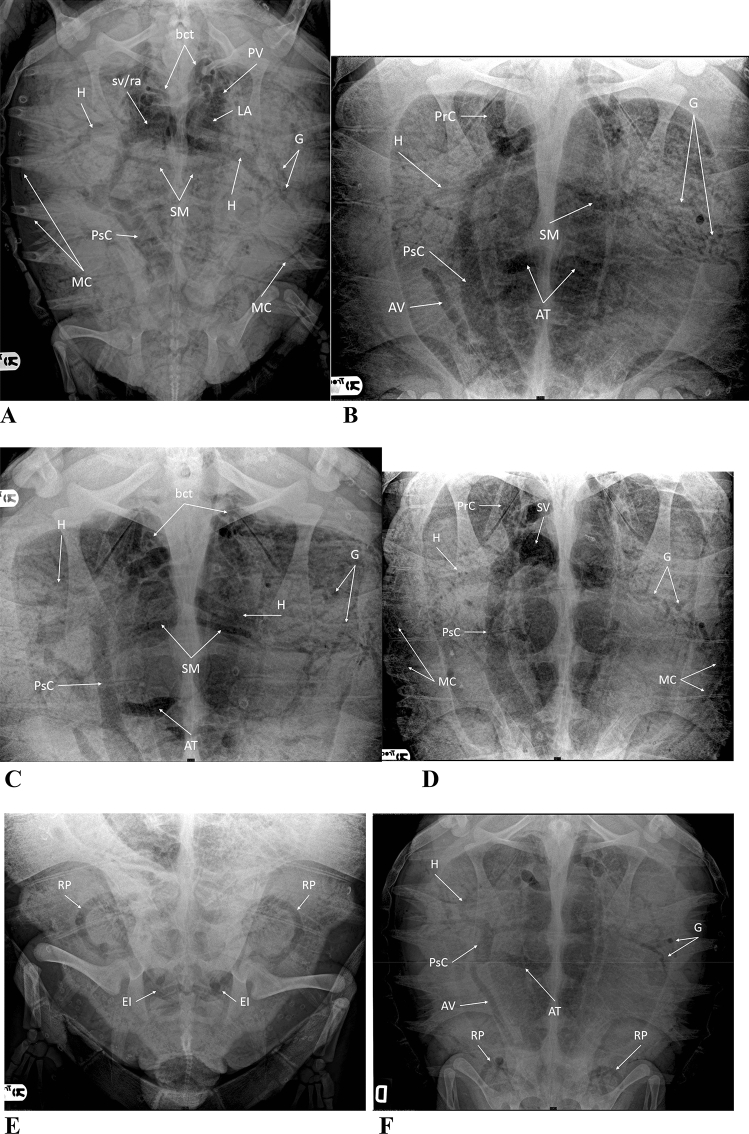
(**A**) Dorsoventral radiograph of a Loggerhead turtle (*Caretta caretta*) with severe GE. Gas is evident in the sinus venosus/right atrium (sv/ra), left atrium (LA), pulmonary vessels (PV), vessels of the brachiocephalic trunk (bct), hepatic vessels (H), gastric vessels (G), postcava vein (PsC), superior mesenteric artery (SM) and marginocostal vessels (MC); (**B,C**) dorsoventral radiograph of two Loggerhead turtles (*Caretta caretta*) with GE of medium to severe degree. Precava (PrC) and postcava vein (PsC), vessels of the brachiocephalic trunk (bct), hepatic vessels (H), gastric vessels (G), superior mesenteric artery (SM), abdominal vein (AV) and transverse abdominal vein (AT); (**D**) dorsoventral radiograph of a Loggerhead turtle (*Caretta caretta*) with severe GE. Gas fills the sinus venosus (SV), left atrium (LA), precava (PrC) and postcava vein (PsC), hepatic vessels (H), gastric vessels (G) and marginocostal vessels (MC); (**E**) dorsoventral radiograph of a Loggerhead turtle (*Caretta caretta*) with GE of mild degree. Gas is present in renal portal vessels (RP) and external iliac vessels (EI); (**F**) dorsoventral radiograph of a Loggerhead turtle (*Caretta caretta*) with GE of medium degree. Gas is present in postcava vein (PsC), hepatic vessels (H), gastric vessels (G), abdominal vein (AV), transverse abdominal vein (AT) and renal portal vessels (RP).

We found 14 cardiovascular sites (CAS) where gas could be detected. Gas distribution within the cardiovascular system was assigned to one or more of these 14 anatomic sites (Table 2, Figs. 4, 5).

**Table 2:** Anatomic sites of gas distribution as seen radiographically in 204 loggerhead turtles affected by GE.

Anatomic site	H	EI	PsC	MC	RP	AV	PV	AT	G	PrC	bct	SM	sv/ra	LA
Number of turtles and percentage of turtles affected at each site	204 (100%)	192 (94.1%)	187 (91.6%)	184 (90.2%)	182 (89.2%)	164 (80.4)	149 (73%)	140 (68.6%)	137 (67.15%)	132 (64.7%)	118 (57.8%)	110 (53.9%)	71 (34.8%)	50 (24.5%)

*H* hepatic vessels, *EI* external iliac vessels, *PsC.* postcaval vein, *MC* margino-costal vessels, *RP* renal portal vessels, *AV* abdominal vein, *PV* pulmonary vessels, *AT* transverse abdominal vein, *G* gastric vessels, *PrC*. precaval vein, *bct* brachiocephalic trunk, *SM* superior mesenteric artery, *sv/ra* sinus venosus/right atrium, *LA* left atrium.

GE involved at least 2 CAS (median 10, IQR 7–13). Among the 40 turtles that died the median number of CAS was 14 (IQR 13–14), while among the 164 survivors the median number of CAS was 9 (IQR 7–11) and the difference was significant (KW = 70.877, p < 0.0001). Applying the ROC curve analysis, it was found that over the threshold of 12 VA there is a high probability of death (AUC = 0.98, SE = 0.0052, p < 0.0001). The risk of death for those with 12 or more VA was 5.91 (95% confidence interval 3.13–11–19).

A logistic regression was applied also to evaluate which CAS was a higher risk of death. Hepatic vessels were deleted both from univariable and multivariable models because all turtles had involvement of that CAS. A very high risk of death was found for GE in the left atrium (OR 40.241, 95% CI 14.8–108.9, p < 0.0001), pulmonary vessels (OR 17.2, 95% CI 2.29–128.81, p = 0.0056), sinus venosus/right atrium (OR 38.47, 95% CI 11.2–132.24, p < 0.0001), abdominal vein (OR 10.97, 95% CI 1.46–82.61, p = 0.0201), transverse abdominal vein (OR 23.37, 95% CI 2.99–137.13, p = 0.0025), and precaval vein (OR 26.62, 95% CI 3.56–198.72, p = 0.0014).

Radiographic examination 65 (32%) of the 204 animals with radiographic signs of systemic GE also showed signs of drowning (Fig. S1). Of the 65 subjects with signs of drowning, 31 (48%) died while 34 (52%) survived. By contrast, of the 278 turtles that did not show radiographic signs of GE, eight (3%) showed radiographic signs of drowning but did not die. In GE-affected turtles drowning signs emerged as a statistically significant factor increasing risk of death in univariable analysis (OR 13.19, 95% CI 5.55–31.34, p = 0.0001). However, the multivariable model with stepwise selection left in the final model only left atrium (OR 22.49, 95% CI 5.81–87.18, p < 0.0001) and sinus venosus/right atrium (OR 24.32, 95% CI 7.95–74.45, p < 0.0001). No other radiographic variables were independently and significantly associated with risk of death.

In the surviving turtles, the gas gradually disappeared radiographically from the different CAS over a period of 2 to 13 days, and the rate of disappearance was related to the severity of the GE. When affected, the heart, venous sinus and great vessels were the first CAS in which the gradual reacquisition of normal radiopacity, a sign of the disappearance of gases, was observed, while the renal and iliac vessels were in all cases the last (Fig. S2).

### Blood analysis results

Blood samples were collected in a time ranging between 30 and 90 min from the arrival of the turtles at the STC. In some cases, during the venous sampling, abundant presence of gas mixed with blood was observed (Fig. S3). PCV ranged from 22 to 44% (mean 33 ± 5.56) and was slightly above normal ranges in about one third of all the turtles with GE analysed, without any statistical correlations with severity or mortality. Blood gas, hematologic and chemical analytes of clinical relevance were obtained for 51 of 184 loggerheads with GE. WBC was within normal range. Creatine kinase (42,130 U/L to 813 mean 1902 U/L) was elevated for all the turtles tested^22^. Phosphorus was higher in turtles that died (median 10.8 mmol/L) than those that survived (median 6.6 mmol/L) (p < 0.0001). No other biochemical analytes were statistically related to mortality.

Results of blood gases analysis in dead and surviving turtles and ROC curve analysis of blood gases biomarker are shown in Tables 3 and 4. The analyte with the highest AUC was pH (AUC = 0.923, 95% CI 0.85–0.99), and the risk of death increased if pH < 7.26.

**Table 3:** Mean of the blood gas results in dead and surviving GE-affected turtles.

	pH*	pCO_2_ (mmlHg)*	tCO_2_ (mmol/L)*	pO_2_ (mmolHg)	Na+ (mmol/L)	K^+^ (mmol/L) *	HCO_3_^−^(mmol/L)	Cl^−^ (mmol/L)
Turtles died n:13	7.17^†^	44.1^†^	23.1	32.30^†^	151.45	4.26	37.1	114.0
Turtles Survived n:38	7.44^†^	32.23	28,16	38.024^†^	154.42	3.27	25.81	115.05

*p < 0.05.

^†^Value out of range^23^.

**Table 4:** Results of the analysis of the ROC curve of each blood gases biomarker in a sample 51 sea turtles with area under the curve (AUC, determined according to the non-parametric method of DeLong and Clarke-Pearson), its 95% confidence interval, the threshold (cut off), and adjusted p value, as mortality predictors.

Blood analyte	Cut off	AUC	95% CI AUC	Adjusted p-value
pH	≤ 7.26^†^	0.923	0.850 to 0.997	0.0006
pCO_2_	> 33	0.863	0.759 to 0.967	0.0006
K^+^	> 3.2	0.772	0.632 to 0.912	0.0006
tCO_2_	≤ 23.4	0.749	0.595 to 0.902	0.0051
SO_2_	≤ 60^†^	0.739	0.530 to 0.947	0.0602
HCO_3_	≤ 24.2^†^	0.726	0.534 to 0.918	0.0592
pO_2_	≤ 21^†^	0.696	0.503 to 0.890	0.0986
Na^+^	≤ 152	0.672	0.484 to 0.861	0.1388
O_2_Ct	≤ 12.1	0.603	0.402 to 0.803	0.4139
Cl^−^	≤ 112	0.575	0.368 to 0.781	0.5428

^†^Value out of range^23^.

A multivariable Cox regression was performed with the following covariates: pH (< 7.26/ > 7.26), pCO_2_ (> 33/ < 33 mmHg), BE (< − 11/ >  − 11 mmol/L), Potassium (> 3.2/ < 3.2 mmol/L), tCO_2_ (< 23.4/ > 23.4 mmol/L), HCO_3_ (< 24.2/ > 24.2 mmol/L), SO_2_ (< 60/ > 60%). Following stepwise regression, only pH was retained in the model as an independent risk factor for death and this means that other factors, significant in the univariable model, could not be considered as factors that independently affect the risk of death (Table S2).

### Post-mortem examination results

Necropsy was completed for 33 turtles. Gas macro bubbles were found in most arterial and venous vessels of all main organs and in major vessels. In these cases, vessels were completely filled with gas even for some centimetres along their length with side-by-side serial macro bubbles showing evident vascular obstruction (Fig. S4). In severe cases, inside the lumen of the precaval vein, there was no presence of blood as it was completely replaced by gas (Fig. S5A,B).

In 12 cases the heart appeared very dilated by intracardiac gas and 20–30 mL of gas were aspirated from each atrium (Fig. S5C,D).

## Discussion

By-catch has become a serious conservation challenge for marine megafauna worldwide and represents one of the most serious threats to sea turtle populations^12,15,24^. To date, relatively few studies with small sample sizes have been conducted on GE in accidentally trawled sea turtles^9,20,21^. The present study provides the largest data set to date for sea turtles affected by GE, including a diverse collection of variables and clinical data. In air-breathing terrestrial mammals, the risk of GE is correlated with dive depth, time at depth, ascent rate (pressure reduction) and temperature^21^. In the current study we were able to examine the full suite of parameters known to correlate with the risk of GE and we have found that all of these parameters also influence the occurrence of GE in sea turtles. Loggerhead turtles can rest on the ocean bottom for over 7 h in cold temperatures^25,26^ but if turtles are struggling in nets, heart rate will increase from vigorous activity, which will result in rapid oxygen depletion. Higher heart rate will increase pulmonary blood flow, which may contribute to increased nitrogen absorption, potentially resulting in the development of gas emboli^27^. However, the exact etiopathogenetic mechanism of this systemic macro embolism in sea turtles has not yet been clarified.

In human scuba diving, GE is associated with breathing gas at increased pressure, which often leads to tissue gas supersaturation during ascent and the formation of venous gas emboli (VGE)^1^. VGE crossover to systemic arteries (arterialisation), mostly through the patent foramen ovale or the intrapulmonary arteriovenous anastomoses, causing a systemic gas embolism^28^. In sea turtles a cardiac right to left shunt occurs during the dive and the consequent apnoea. Besides intracardiac shunts, pulmonary-to-systemic shunting has been described between the pulmonary arteries and veins suggesting control of perfusion at several levels^29,30^. Recently multiple physiological arterio-venous anastomoses were detected between pulmonary arteries and veins on high resolution Multidetector Computed Tomography images^31,32^. We hypothesise that all these shunt mechanisms play a role in the systemic blood diffusion of the gas during the entrapment of turtles in fishing nets probably starting from the pulmonary circulation. In systemic GE in turtles, the gas is present throughout the venous and arterial circulation because the gas may pass from the heart or arteriovenous anastomoses from the venous to the arterial compartment. It is plausible that the significant amount of gas found throughout the venous and arterial circulation and in the heart causes a slowing or even an arrest of the blood circulation. Nevertheless, “only” 20% (40/204) of the turtles with GE in the present study died and many animals with severe disease spontaneously eliminated the gas bubbles and recovered. Considering the high amount of gas present in the cardiovascular system of turtles affected by GE, pathologically this disease could be compared to the massive systemic air macro embolism of humans a rare but catastrophic and largely underdiagnosed, undertreated, and underreported disease^33^. The brain and heart are the end organs most vulnerable to these ischemic events, which can lead to irreversible sequela or death. The air emboli cause pathological changes by two mechanisms: a reduction in perfusion distal to the obstruction and an inflammatory response^34^ with platelet and leucocyte aggregation, cytokine release, and activation of the complement, fibrinolytic and coagulation cascades. This subsequent acute inflammatory response results in increased capillary permeability mediated by endothelial damage, oedema and haemoconcentration^35^. These processes lead to vasogenic oedema worsening the ischemia of the end organs^36^. We found peripheral oedema in 95% of the turtles with GE presumably due to mechanical obstruction of vasculature by bubbles and acute inflammatory response. Unlike what has been reported recently^20^ where PCV was significantly lower in turtles that died from GE, we found no statistically significant PCV alteration in the turtles that died. Seven turtles with GE showed retraction of the hind limbs under the carapace but we must consider that the hind limb retraction under the carapace is also a frequent sign of severe stress in sea turtles and it is considered a normal protective rear flipper clasp reflex^37^.

One of the goals of the study reported here was to document venous blood gas and acid–base values for loggerhead turtles with GE 5–7 h after trawling and to investigate differences in these values between turtles that survived and those that died. In turtles with GE the blood circulation is dramatically slowed or even stopped due to the gas emboli disseminated along the cardiovascular system, and peripheral tissue O_2_ extraction should be considerable. In the present study pH, pCO_2_, tCO_2_, HCO_3_, SO_2_ and potassium concentration were found to be relevant in the clinical assessment of turtles affected by GE but only pH was an independent risk factor for death. Acidemia was certainly related to inability to breathe and poor tissue perfusion due to GE. Our data agree with a number studies that have demonstrated that trawl-captured loggerheads exhibit a marked acidemia and lactic acidosis^38,39^. In several studies the resolution of acidemia was accompanied by a decrease in pCO_2_ with a respiratory rates considerably above reported rates for unrestrained sea turtles^38–40^. Respiratory compensation, not an option during trawl captures, appears to be critical in maintaining physiologic blood pH^39^. We found that respiratory rates did not differ significantly between turtle that survived and those that died, though respiration was lower in all the GE turtles than reported respiratory rates of loggerhead sea turtles after 30 min on-board recovery following capture by trawl net (trawl T_0_, median 5.2, range 1.0–10 breaths/min). Respiratory rates were similar to captive swimming unrestrained subadult loggerheads at 22–25 °C (0.34 breaths per min)^40^. This certainly affects the ability of sea turtles with GE to correct severe acidemia.

Furthermore, the turtles with GE had low body temperature (median 12.5 °C) and in the evaluation of the HCO_3_ it must be considered that in sea turtles the reduction of body temperature determines an increase of HCO_3_. Temperature-related adjustments of blood pH in sea turtle appear to be managed at both lung and tissue (ion exchange) levels and bicarbonate is not consumed by acid titration, but rather that transcellular ion exchange processes using HCO_3_ are implicated^41^.

Sea turtles continue to survive up to 5 h after there is a total absence of oxygen in blood and lungs^42^. Loggerheads also maintained electrical activity of the heart for over 1 h of total anoxia^43^ in very sharp contrast to mammals. Turtle brain ATP levels are maintained for at least 2 h of anoxia^44^ while depletion occurs within minutes in mammals. During extended dives all tissues may become anaerobic and large changes in blood pH and pCO_2_ are tolerated^45^. The major adaptation that allows turtles to endure total anoxia for many hours is the special ability of the brain to function in the absence of O_2_.

Hyperbaric oxygen (HBO) is the main therapy for massive gas embolism from any cause because it may not only decrease the size of air bubbles but also provide an adequate supply of oxygen to the ischemic tissue, but such a treatment can only be performed with specialised equipment^46^. There is no evidence that treatment with corticosteroids, anticoagulants, or lidocaine is related to a beneficial outcome in human massive gas embolism^34^. Moreover, in turtles with severe GE, initially the blood flow is slowed or even completely stopped so we do not know if the parenteral administration of any drug can be effective. In fact, the results of the post-mortem examination allowed us to confirm the radiographic findings, as we detected the heart and vessels completely filled with serial macro gas bubbles causing an evident vascular obstruction.

Theoretically, gas embolism in turtles must be treated in a hyperbaric chamber as soon as possible but studies on large numbers of animals must be carried out to understand if it is promptly effective on turtles with very severe GE, destined to die within a few hours (most of the turtles with severe GE of the present study died within 6–8 h).

In previous studies on lower numbers of turtles accidentally caught by trawls and gillnet, the mortality caused by GE was between 41 and 30%^9, 21^. These turtles died during hospitalisation in a time comparable to that observed in our study. In a recent study^20^ 12 of 28 (43%) animals died on-board fishing vessels and 3 of 15 (20%) active turtles released with satellite tags died within 6 days. About 90% of turtles with GE, alive upon arrival at STC, recovered from the disease in a time ranging from 2 and 13 days without any supportive drug therapy but only kept *dry-docked* or in tanks with low water level in a room at approximately 25–30 °C.

According to several studies^17,18^, direct mortality due to trawling depends on tow duration and hence the submergence time. Interestingly, in the multivariable analysis of the current study, drowning is not a risk factor for death, but the only risk factors are the number of the CAS and the presence of GE in the left atrium and sinus venosus/right atrium. According to these data it can be deduced that trawled sea turtles rarely undergo drowning if not also suffering from GE and that mortality is closely associated with the presence of drowning contextually to GE. Specifically, we can therefore hypothesise that the animals probably first undergo severe GE involving the heart and only then drown. In a recent study, GE entails a worse prognosis if it occurs concurrently with water aspiration^20^. In our opinion therefore, trawling turtles essentially die of severe undiagnosed GE which is related to depth, duration of the trawling, ascent rate at which turtles were caught as well as cold season fishing, and water aspiration associated with forced submergence is not the leading cause of death. However, the results of the present study show that the statistically significant predictive factor of mortality related to trawling, is exclusively the ascent rate of the nets.

Intense loggerhead turtle interactions with trawl nets have previously been described in the Adriatic Sea^46–49^. This area is characterised by shallow waters (< 100 m) and rich benthic communities where turtles in the demersal stage spend the winter^16,25,46–49^. Mediterranean loggerhead sea turtles increase time of submergence and rest on the bottom during the coldest periods of the year. In this season, bottom trawling mostly interferes with the demersal stage of the loggerheads that are more likely to assemble in shallow water in order to feed on abundant benthic and epibenthic prey^47,48^. Furthermore, this seasonal difference is partially due to a higher level of catchability at low temperatures, when turtles seem to be slower in avoiding nets than they are at higher temperatures^25,46,47^. Turtles in the present study were trawled mostly in the winter with juveniles comprising over 60% of the bycaught animals. After April in the southern Adriatic Sea, turtles accidentally caught by trawling are almost never observed. The highest rate of bycatch in Valencian coast of Spain occurred between November and March when most GE cases were encountered^9^.

In the present study, radiographic examination proved to be an excellent diagnostic tool to evaluate GE in sea turtles. Full-body radiographs in the DV projection are easy to perform and allow effective diagnostic evaluation because it is possible to observe all the defined CAS affected by the accumulation of gas. The DV projection is helpful for identifying pathological radiopacity of the lungs as in drowning, but the cranial-caudal and LL projections were more useful to view the lungs without the overlap of celomic soft tissues. The LL projection is useful for the evaluation of the renal CAS (external iliac and renal portal vessels), which however are also evident in the dorsal–ventral projection, and severe accumulation of gas in the heart. As with the liver, the presence of gas in the external iliac and renal portal vessels is not significant for the prognosis because gas in these vessels is present in almost all GE cases (94% and 90% respectively). Compared to ultrasound, the radiographic examination allows a better overview of all the CAS with less stress for the animal. In our experience, because 12/14 CAS were a statistically significant risk factor associated with mortality, it is important to be able to observe all the CAS through the radiographic examination. The advantage of ultrasound is that sea turtles can be examined on-board fishing vessels, but in animals larger than 30 cm length (CCL), many areas, such as the heart or major vessels, cannot be examined^20^. In the present study, high risk of mortality was identified just for these CAS (left and right atrium, pulmonary vessels, sinus venosus, and major vessels) and the median CCL of the turtles was 64 cm. Therefore, in most cases radiographic examination is essential for a complete evaluation of the lungs and the CAS and then to define the severity of the disease. Ultrasonography for animals larger than 30 cm in length is useful as a screening to evaluate if they suffer from GE because the renal vessels are involved in more than 90% of the cases and are always easily identifiable^9, 20^. During the radiographic follow-up of the turtles with GE we observed that gas in the renal vessels is also the last to disappear, so ultrasonography can be useful to monitor resolution of GE.

There has been still limited study and with small sample size of turtles for post-release mortality of marine turtles following capture by trawl fisheries^20,50,51^. Parga et al. described that on 15 animals that survived and were released with sPAT (survivorship Pop-up Archival Tag) tags three (20%) died during the first 6 days and the outcome of a fourth animal was unknown. The immediate release of turtles into the sea can only be an option if there is no rescue centre available to refer the turtles to. On board the fishermen cannot stage the disease and understand its severity. Comatose turtles cannot swim and may therefore be unable to surface to breathe if released into the sea in this condition^47^. From the statistical data of our study, only turtles in a comatose state are obviously serious, but turtles without pathognomonic clinical signs can also be severe and RX staging is always required. Except animals that died within the first 12 h of hospitalization, all the animals in our study underwent radiographic checks every 48 h with progressive improvement of the gas inside the vessels and clinical improvement. In no animal have we observed worsening in quantity of gas in the following days.

In light of the results achieved in this research, it is strongly recommended to transport the turtles as soon as possible to rehabilitation or rescue centres, rather than releasing them directly into the sea, even after a resting phase; in fact, turtles that apparently appear to be in good condition may actually have a GE in progress. Waiting to understand how to stem the phenomenon during fishing, collaboration with the fishermen is essential because it is important that the sea turtles are immediately transported to the rescue centres in order to be housed in the tanks in case of GE so as to control and to reduce their mortality.

The adoption of conservation actions has become a strategic issue in the Mediterranean, where the commercial fishing appears to be the main driver of mortality for marine turtles^15,49,52^. Therefore, modifications to fishing gears and practices are urgent to prevent sea turtle population collapse in this basin. However, these changes should fit some simple requirements to be accepted by fishermen and to be effective: they should be practical at sea (do not involve major changes to the common practices, easy to use and cheap to maintain), acceptable for fishermen (economically viable), acceptable for management (achieves the management and biological targets), enforceable (easy to be controlled by fisheries inspection bodies). In bottom trawling the most effective solution to reduce GE phenomena is to limit the residence time of turtles inside the net, once incidentally captured. This can be achieved by introducing technological improvements in fishing gear, the so-called Bycatch Reducer Devices (BRDs) as the Turtle Excluder Device (TED) that seems to be the most effective. In the last 6 years, the effect of a new prototype of TED, a flexible grid, on the catching efficiency and performance of commercial bottom trawl has been tested in several bottom trawlers in the Adriatic Sea^53,54^. Easy storage and handling compared with previous devices tested in this area^55^ make the flexible TED a practical and valuable solution to reduce turtle bycatch in coastal Mediterranean demersal multispecies fisheries, without negatively affecting the capture of commercial species. Because of their effectiveness, which has mainly been demonstrated in prawn trawl fisheries, TEDs have become mandatory in several countries outside the Mediterranean. Unfortunately, despite BRDs being an increasingly urgent need, they are not accepted in any Mediterranean country, because conservation aims are often hampered by competing political and economic factors^24^. Therefore, to make the adoption of TEDs (and BRDs in general) acceptable to fishermen, a feasible measure could be that of quality labels, which could allow to add value to the fish caught sharing the principles contained in a procedural guideline. The results obtained in the present study suggest to include in the guidelines the commitment by fishermen to reduce the fishing depths and the speed during the net hauling and to limit the haul duration within 4 h of fishing, at least during the critical winter periods.

In conclusion, besides to applying the BRDs and outreach programs, the measures to reduce the impact of bycatch consist of raising fishers’ awareness and in training them in the best practices in sea turtle recovery after capture. We deem that the conservation of sea turtles is mainly a technical and political challenge, because all these measures require significant investments but are capable of improving the conservation prospects of these endangered species^19^. Such efforts would so be designed to reach the UN Sustainable Development Goals of 2030 Agenda, particularly the targets of Goal 14^56^, and in the context of the European Union Green Deal and the recent Recovery and Resilience Facility.

Currently, the synergy between fishermen, rescue and rehabilitation centres is important to reduce mortality from systemic GE in sea turtle, because 80% of all the affected animals will have a good chance of survival and be released, if kept in shallow water or dry for at most 2 weeks needed to recover from systemic macro embolism.

## Methods

Between January and April 2016 and October 2016 and February 2017, loggerhead sea turtles incidentally captured (bycaught) in trawls along the Italian coast of the South Adriatic Sea were admitted to the Sea Turtles Clinic (STC) at the Department of Veterinary Medicine in Bari (Italy) for veterinary evaluation after having been taken to the local Adriatic Sea turtle rescue centre (World Wildlife Found, WWF, Molfetta).

The fishermen, sensitised by the local sea turtle rescue centre, alerted the rescue centre staff via a WhatsApp message as soon as they accidentally caught the turtles. After finishing the fishing trip, they sent a second message shortly before arriving to port to make the rescue centre staff ready to immediately transfer the turtles to the STC. The information relating to depth and duration of trawling, ascent rate (m/min) and any symptoms noted on board the vessels were provided by the fishermen to the rescue centre staff and reported to the STC at the time of admission of the turtles.

### Clinical and radiographic evaluation

Immediately upon admission, physical examinations were performed of each turtle, including curved carapace length, curved carapace width, and weight, as well as core body temperature that was measured via a temperature probe inserted ~ 10 cm into the cloaca.

Full-body radiographs in dorsal–ventral (DV vertical beam) (Eurocolumbus, AEX 125 kV/300 Ma) and cranial-caudal (Cr-Cd horizontal beam) and lateral-lateral (LL horizontal beam) (Diagnostic X-RAY unit, Orange 1060 HF Ultra Plus) projections were performed. All radiographs were assessed independently by three experienced sea turtle veterinarians (A. D. B., D. F. and C. V.) to evaluate signs of drowning, characterised by interstitial and peribronchial thickening, and gas embolism within the cardiovascular system. In all turtles with radiographic signs of GE, we evaluated and listed the cardiovascular sites (CAS) where the gas was well detectable.

After radiographic examination, in turtles with signs of GE, respiratory rate (assessed visually), heart rate (assessed by ECG), PCV, and neurological and sensory deficits were recorded. Mentation was assessed as alert (responsive to external stimuli), depressed (reduced, compared with normal, but appropriate responsiveness to external stimuli) and lethargic/comatose (not responsive, loss of consciousness)^57^.

Heart rate was performed with the turtle placed in the transport tank, after a 10 min period from the previous manipulations, using ECG vector in lead II with electrodes distributed on the four limb positions^58^. Time to perform ECG was ranged from 3 to 5 min.

Additional clinical findings that were recorded included peripheral oedema such as neck or palpebral oedema, cloacal prolapse and the eventual presence or absence of particular postures such as retraction of the hind limbs under the carapace.

### Blood analysis

Between January and February 2017 blood samples for hematologic and biochemical analyses were collected from loggerheads affected by GE immediately after the radiographic diagnosis. Venous blood samples were collected anaerobically from the external jugular vein into a heparinized 2.5 mL syringe and processed immediately. Whole blood was used for determination of PCV, leucocyte count (WBC), and blood gas and electrolyte analysis. Plasma was then harvested from the remaining sample after centrifugation. PCV was measured after centrifugation of blood at 13,000×*g* for 5 min in haematocrit tubes. WBC was performed manually by use of a disposable pipette system^58^. Blood gas and electrolyte analysis included pH, pCO_2_, HCO_3_^-^, pO_2_, SO_2_, anion gap, tCO_2_, sodium, potassium and chloride (IDEXX VetStat Electrolyte and Blood Gas Analyzer, IDEXX VetStat Respiratory/Blood Gases, IDEXX Cassette Respiratory/Blood gases, IDEXX laboratories, Inc). Plasma biochemical values were measured by use of an automated clinical chemistry analyser (Beckman Coulter, AU680). Blood gases and pH were corrected for the patient’s body temperature: values for pH, pCO_2_, and pO_2_ were corrected for the patient’s body temperature and ionized calcium was corrected for pH using published equation^59^. Bicarbonate concentration was calculated using the Henderson–Hasselbalch equation, temperature-corrected pH and pCO_2_.

### GE management

Turtles with radiographic evidence of GE were kept in padded boxes out of the water (“dry-docked”) or in tanks with low water level in a room at approximately 25–30 °C until clinical improvement. GE-affected turtles did not receive any pharmacological treatment. The animals were clinically monitored daily, while blood analysis and radiographic examination were performed every 48 h until complete resolution of clinical or radiographic evidence of GE, or until eventual death.

### Post-mortem examination

Necropsies were performed within 12 h after retrieval from fishing gear or within 3–4 h following death at STC. Systematic sea turtle necropsy procedures were followed^60^. The carcass was positioned in dorsal recumbency and carefully removed the plastron, minimising artefactual gas infiltration by traction of tissues and during sectioning of blood vessels. We first observed the heart in situ then we opened the pericardial sac to inspect the heart and when it was full of gas, we aspirated it with a 20 mL syringe. The major blood vessels and the entire coelomic cavity to evaluate organs for emboli and lesions were subsequently inspected. The last organs examined were the respiratory system, where we checked for froth and fluid, the kidneys and genital tracts.

### Statistical analysis

Qualitative variables were summarised as counts and percentages and comparison between independent groups were performed by chi-square or Fisher exact test as appropriate. Quantitative variables were described as median and interquartile range (IQR) because they did not approach Gaussian distribution (verified by Shapiro–Wilk test) and comparison between independent groups were performed by non-parametric tests (Kruskal–Wallis or U-Mann–Whitney as appropriate for independent samples). Radiographic signs of GE and drowning were tabulated to determine the number of affected anatomic sites, and to determine which sites were correlated with mortality. To evaluate the effect of each cardiovascular anatomic sites (CAS) for death, a logistic regression analysis was performed with the death event as dependent variable and the presence of GE in each CAS as independent variable. The univariate regression was carried out for each CAS and multivariate regression was performed with all CAS included in the model. A stepwise selection was performed to find the best fit. In the multivariable analysis, drowning signs were entered as another explanatory variable. The odds ratio and its 95% confidence interval are reported.

A ROC curve analysis was performed to evaluate the number of CAS involved that relate to a death event, and the area under the curve and its 95% confidence interval, and the threshold values of the number of CAS were determined. Once the cut-off value was determined, the number of CAS was classified in two ways: over and below the cut-off, so a logistic model was applied to quantify risk of death related to number of CAS, and through the calculation of odds ratio (OR) and its confidence interval.

On a sample of GE-affected turtles blood gas parameters were available and a ROC curve analysis was performed to evaluate the diagnostic performance of quantitative variables of blood analytes, with area under the curve (AUC, determined according to the non-parametric method of DeLong and Clarke-Pearson)^61^, its 95% confidence interval, the threshold (cut off), and adjusted p value, as mortality predictors. To evaluate the risk of death related to blood gas and electrolytes variables, classified as over/below the cut-off, a Cox model was used with time to death as outcome. A univariable and a stepwise multivariable Cox model were performed, reporting hazard ratio (HR) and its confidence interval. A p-value < 0.05 was considered as statistically significant. If appropriate for multiple testing procedure, the adjustment of p-value was performed with the FDR (false discovery rate) approach. Analyses were performed with SAS 9.4 and Medcalc 16.2.1.

### Ethical statement

Animal care was applied within institutional guidelines. This study was approved by the ethical committee of the Veterinary Medicine Department of Bari (CESA DiMeV Bari). Clinical information generated for this study was derived from the regular veterinary procedures provided in order to establish an appropriate diagnosis and prognosis in sea turtles. All activities related to veterinary evaluation of bycaught turtles in this study were conducted to provide appropriate care and maximize survivorship. No procedures were conducted solely for research purposes.

## Supplementary Information

Supplementary Information.
