# Supplementary material for: Analysis of risk factors associated with gas embolism and evaluation of predictors of mortality in 482 loggerhead sea turtles
Source: Sci Rep. 2021 Nov 22;11:22693. doi: 10.1038/s41598-021-02017-4 (PMC8608947; doi:10.1038/s41598-021-02017-4)
Supplement: Supplementary file 1 — Supplementary Information. [file 41598_2021_2017_MOESM1_ESM.docx]

**SUPPLEMENTARY MATERIAL**

**Table S1:** Results of the univariable logistic regression and of the multivariable logistic regression with death event as dependent variable. In the univariable model each factor was evaluated alone; in the multivariable all factors were analyzed together but a stepwise method selected the statistically significant factors. Not all significant factors in the univariable were statistically significant in the multivariable.

|  |  | **Univariable logistic regression** | | | **Stepwise logistic regression** | | |  |
| --- | --- | --- | --- | --- | --- | --- | --- | --- |
| **Variable** |  | **OR** | **95% Confidence Interval** | **p-value** | **OR** | **95% Confidence Interval** | **p-value** |  |
| Duration of fishing | as continuous | 1,51 | 0,89 to 2,55 | 0,1222 | not entered | | |  |
| Depth of fishing net | as continuous | 1,018 | 0,998 to 1,038 | 0,0735 | not entered | | |  |
| CCL | as continuous | 0,977 | 0,93 to 1,03 | 0,3453 | not entered | | |  |
| CCW | as continuous | 0,973 | 0,921 to 1,027 | 0,3166 | not entered | | |  |
| Weight | as continuous | 0,977 | 0,945 to 1,01 | 0,1682 | not entered | | |  |
| Gender | F vs M | not estimable | | 0,981 | not entered | | |  |
|  | J vs M | not estimable | | 0,9775 | not entered | | |  |
| Temperature | as continuous | 1,037 0,863 to 1,246 | | 0,6994 | not entered | | |  |
| Respiratory Rate | as continuous | 0.92 0,78 to 1,08 | | 0,2894 | not entered | | |  |
| Peripheral edema | Yes vs No | 2 | 0,24 to 16,39 | 0,5193 | not entered | | |  |
| Cloacal prolapse | Yes vs No | 0,98 | 0,37 to 2,58 | 0,9694 | not entered | | |  |
| Hind limbs under carapace | Yes vs No | 11,63 | 2,16 to 62,5 | 0,0043 | 21,28 | 1,49 to 333,33 | 0,0242 |  |
| Neurological deficit | Yes vs No | 24,39 | 6,76 to 90,91 | <0,0001 | 7,87 | 1,31 to 47,62 | 0,0243 |  |
| Sensory | Comatose vs Alert | 29,78 | 6,13 to 144,76 | 0,0001 | 80,13 | 8,25 to 778,09 | 0,0003 |  |
|  | Depressed vs Alert | 1,67 | 0,65 to 4,3 | 0,0437 | 6,023 | 0,97 to 37,24 | 0,6019 |  |
| PCV (reference value <0.29) | >0.35 | 1.78 | 0.45 to 7.13 | 0.6059 | not entered | | |  |
|  | 0.33 – 0.35 | 1.25 | 0.29 to 5.31 | 0.7511 |  |  |  |  |
|  | 0.29 – 0.33 | 1.92 | 0.51 to 7.29 | 0.4640 |  |  |  |  |
| Heart rate | <=12 bpm vs >12 bpm | 12.69 | 2.82 to 57.07 | 0.0009 | not entered | | |  |

**Table S2:** Summary of predictors of mortality and GE development

| **Predictors of mortality** | |
| --- | --- |
| Ascent rate (m/min) | ≥3.5 |
| CCL (cm)  Heart rate | >64  <12 |
| CAS | >12 + LA, sv/ra |
| pH | ≤7.26 |
| pCO2 (mmHg)*  BE (mmol/L)*  K^+^(mmol/L)*  tCO2 (mmol/L)*  HCO3 (mmol/L)*  SO2 (%)* | >33  <-11  >3.2  ≤23.4  ≤24.2  ≤60 |

* Significant in the univariable model

| **Predictors of GE** | |
| --- | --- |
| Ascent rate (m/min) | >2,5 |
| Trawl Duration (hr) | >4 |
| Trawl Depth (m) | >49.5 |
| Turtle temperature (°C) | <12.5 |

**Fig. S1**. Radiographic study of a Loggerhead turtle (*Caretta caretta*) with GE associated with drowning. **A**) Dorsoventral view shows presence of gas inside the sinus venosus/right atrium (sv/ra), left atrium (LA), brachiocephalic trunk (bct), pulmonary vessels (PV), precava (PrC) and postcava vein (PsC), hepatic vessels (H) and gastric vessels (G). The massive accumulation of gas in the heart chambers and in the brachiocephalic trunk (black arrow) is also evident in the lateral view (**B**). In the craniocaudal (**C**) and lateral projections is evident diffuse opacification of the pulmonary parenchyma, in the dorsal cranial area (empty black arrows) and around the bronchi (empty white arrows), due to the inspiration of sea water.


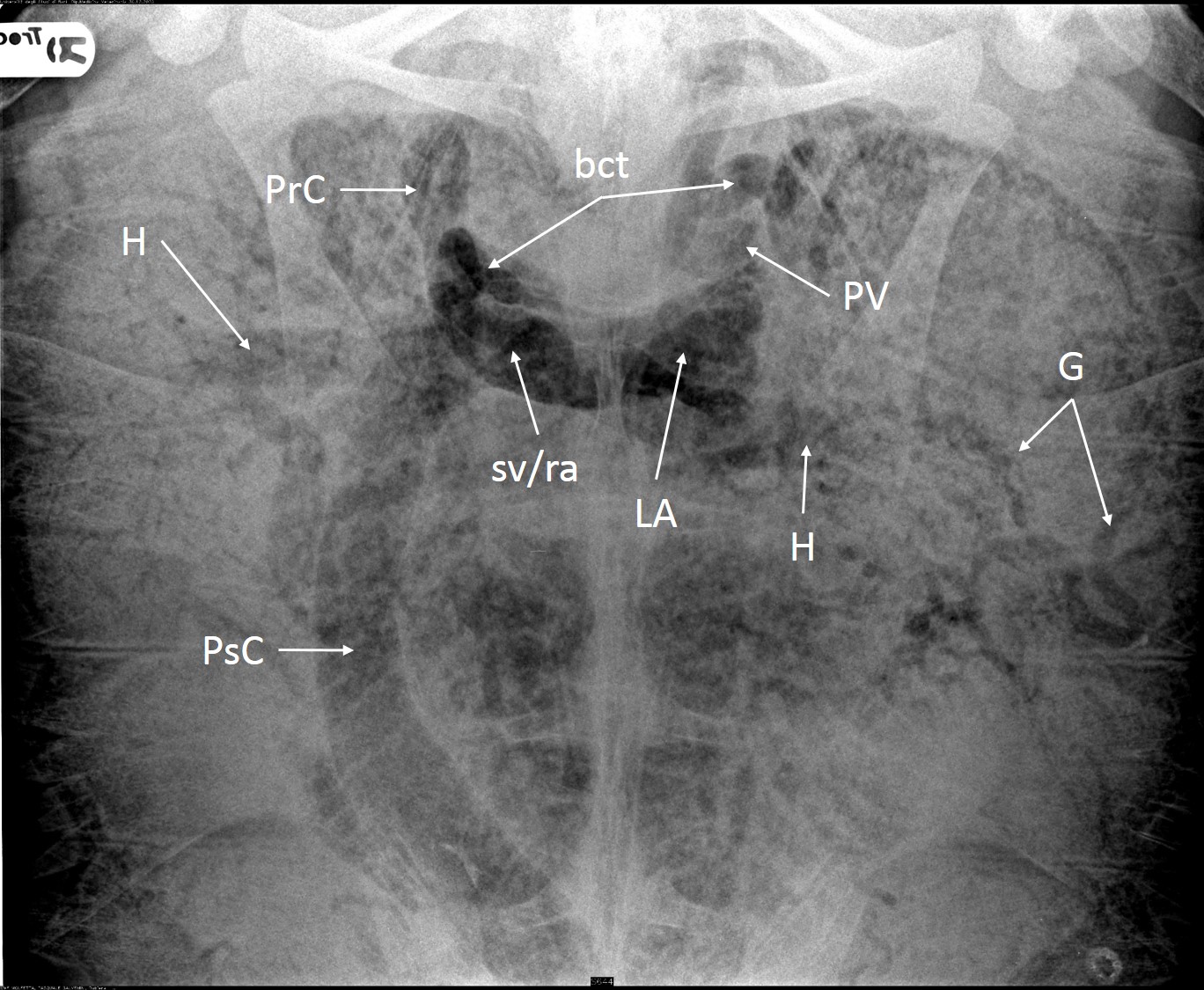

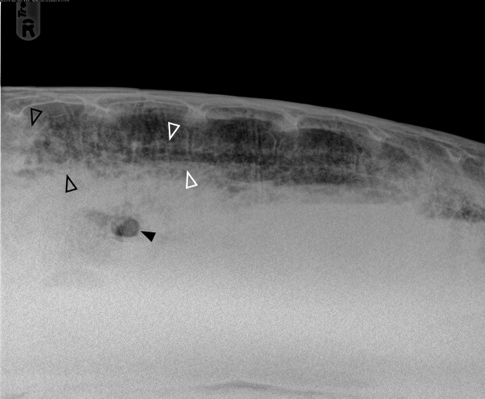

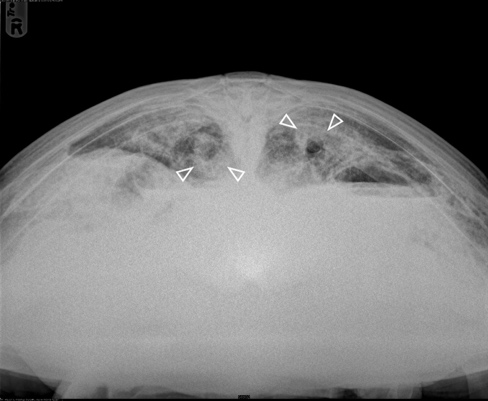


**A B C**

**Fig. S2.** Example of radiographic resolutions in Loggerhead turtles (*Caretta caretta)* with GE. Severe GE **A-D**) days 0,3^rd^ ,6^th^ and 8^th^. Severe GE (**E-G)** days 0, 2^nd^ and 8^th^. Mild GE (**H-I**) days 0 and 3^rd^.

**
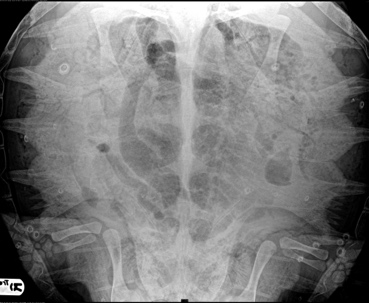

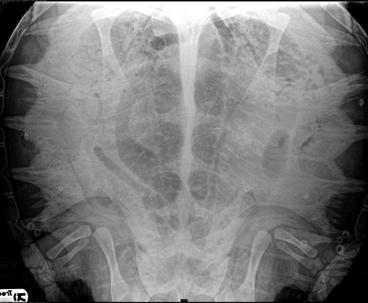

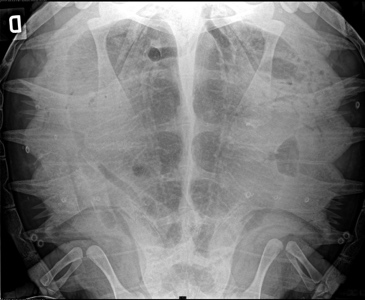

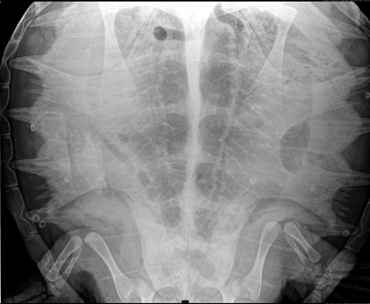
**

**A B C D**


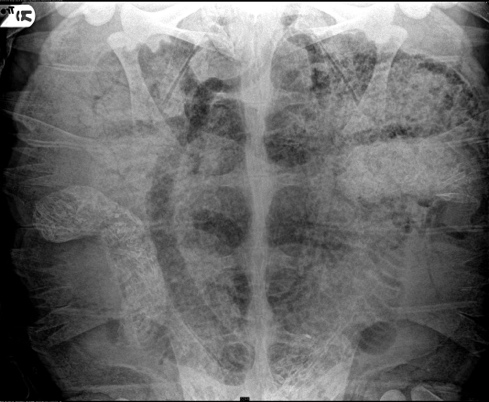

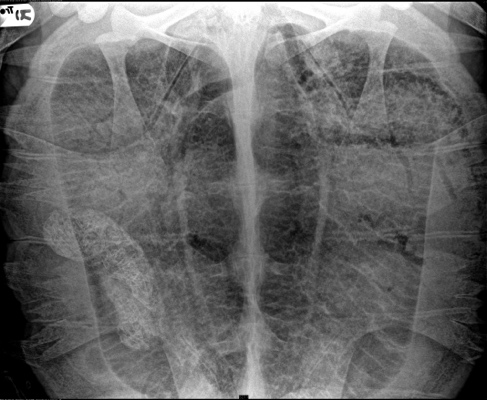

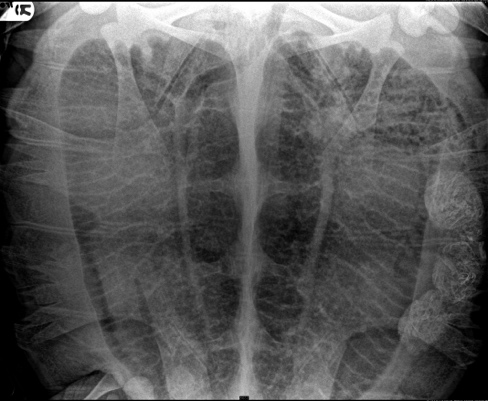


**E F G**

**
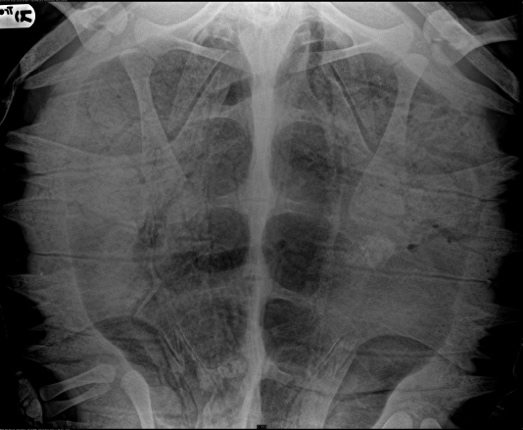

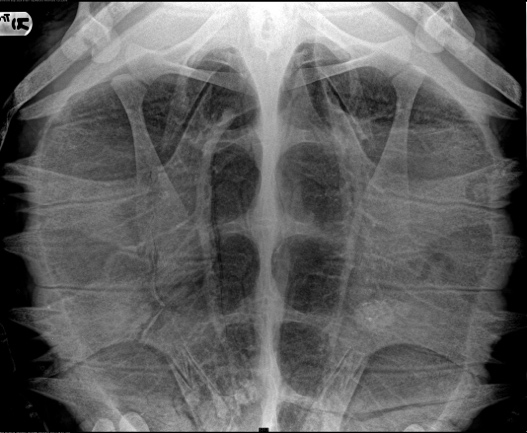
**

**H I**

**Fig. S3.** Example of sampling from the jugular vein in a turtle with GE. Inside the syringe there is blood mixed with air bubbles.


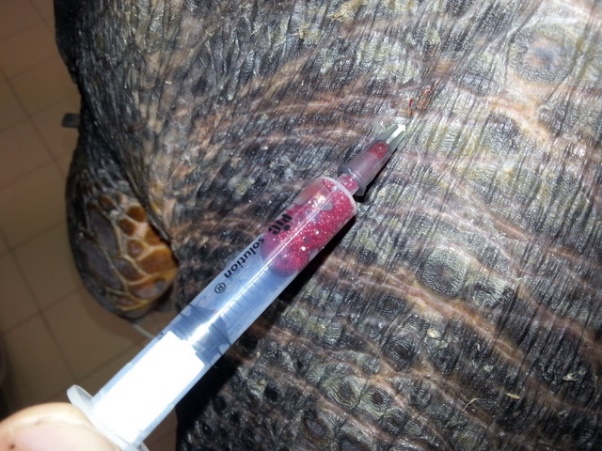


**Fig. S4.** Postmortem examination of a Loggerhead with severe GE: the vessels of the hepatogastric ligament show side by side serial macrobubbles with evident vascular obstruction.

**
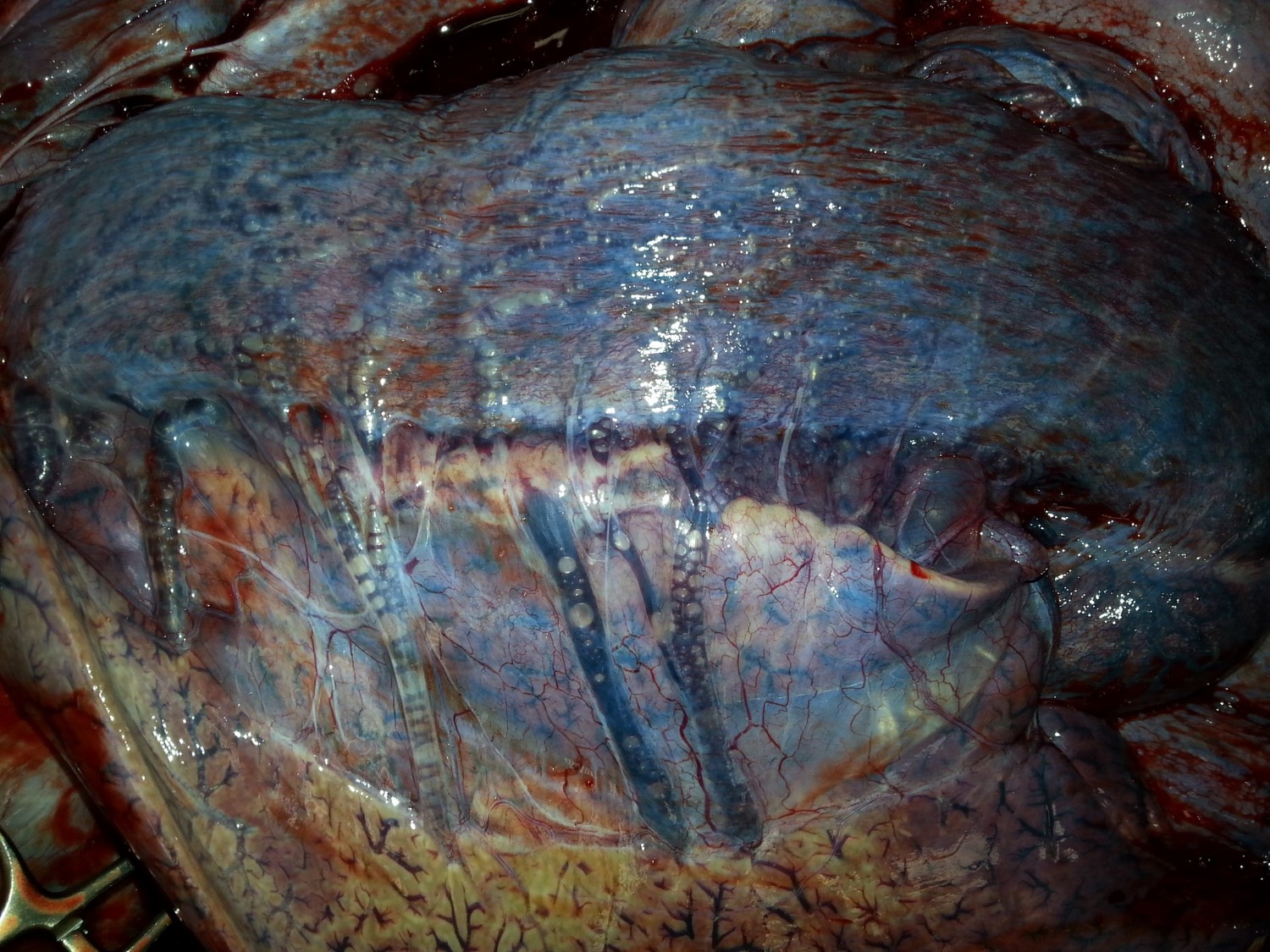
**

**Fig. S5.** Postmortem examination of the heart of a turtle with severe GE after the opening the pericardial sac. (**A**) the right atrium is displaced with forceps to show the precaval vein below that is diffusely distended by gas that completely replaced the blood. **B**) The right and left atrium are markedly distended with intracameral gas. **C)** the gas was aspirated with a 60 ml syringe from the left atrium **D**) the atrium appearance after the air aspirated (20 ml) resembles a “deflated balloon”

**
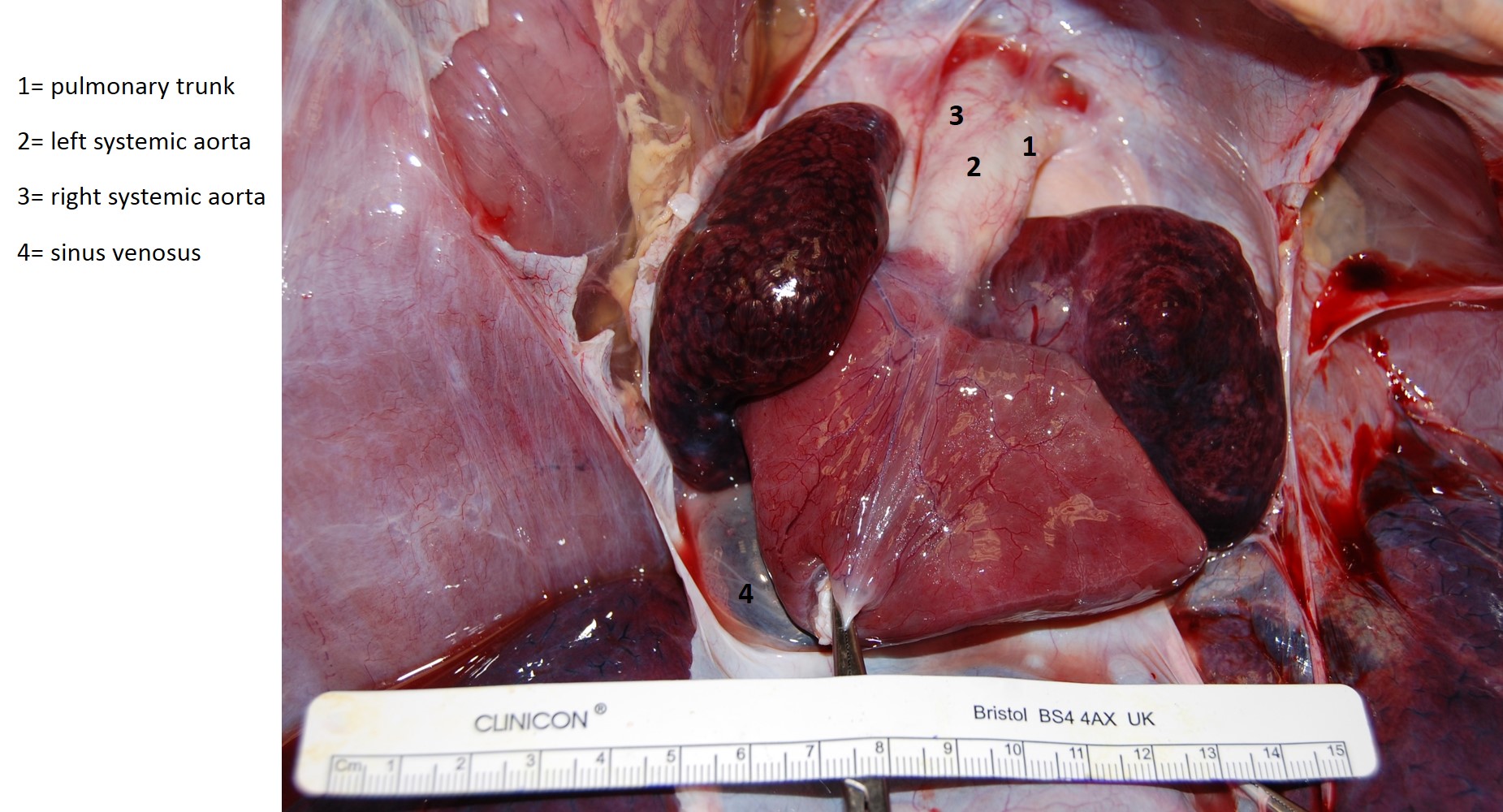
** **
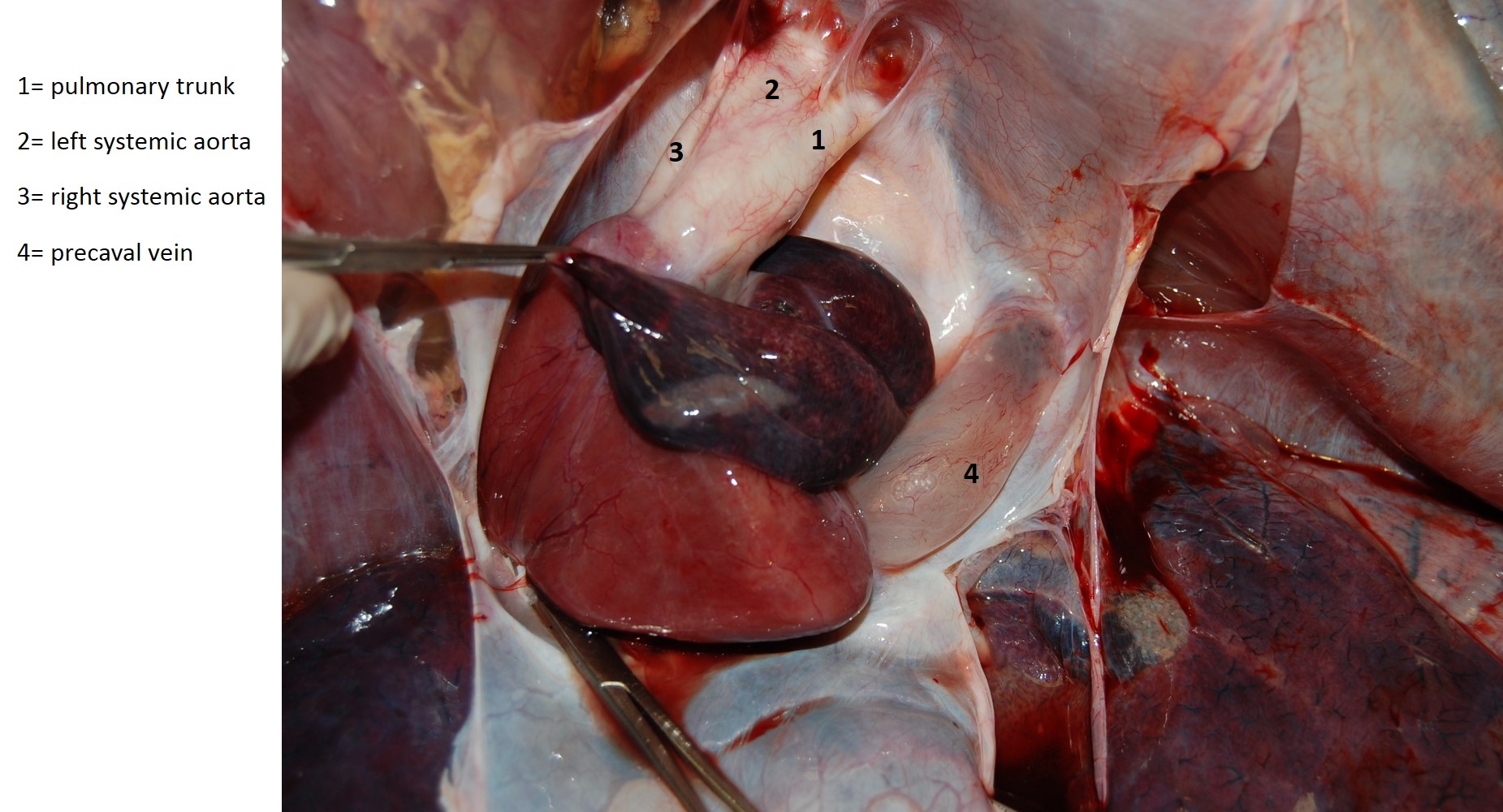
**

A B


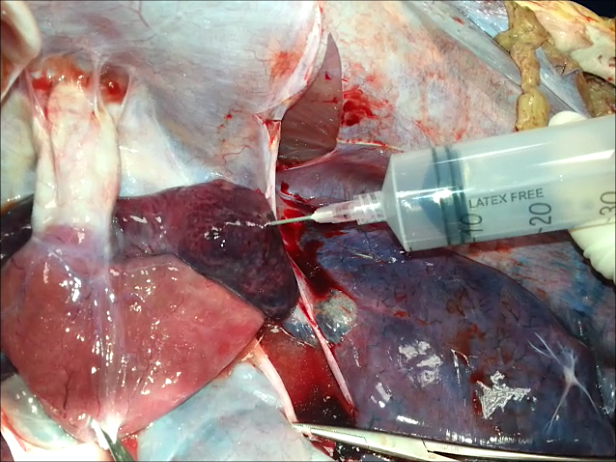

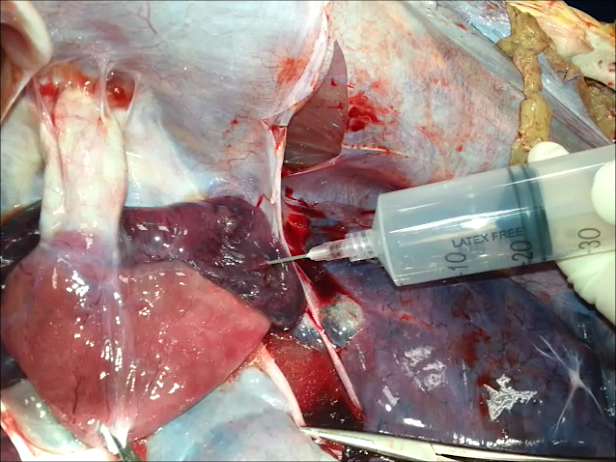


C D
